# Decision-making on colorectal cancer screening in Curaçao - interviews with the target population

**DOI:** 10.1186/s12889-023-16335-x

**Published:** 2023-07-27

**Authors:** Shacara N. Blake, Jacqueline G. Hugtenburg, Manon van der Vlugt, Evelien Dekker, Mirjam P. Fransen

**Affiliations:** 1 Caribbean Prevention Center (Fundashon Prevenshon), Willemstad, Curaçao; 2 Department of Gastroenterology and Hepatology, Amsterdam UMC location University of Amsterdam, Amsterdam, The Netherlands; 3 Amsterdam Gastroenterology, Endocrinology and Metabolism (AGEM), Amsterdam, the Netherlands; 4 Faculty of Social and Behavioral Sciences, University of Curaçao, Willemstad, Curaçao; 5 Department of Clinical Pharmacology, Amsterdam UMC location Vrije Universiteit Amsterdam, Amsterdam, the Netherlands; 6 Department of Public and Occupational Health, Amsterdam UMC location University of Amsterdam, Amsterdam, The Netherlands; 7 Amsterdam Public Health Research Institute, Amsterdam, the Netherlands; 8 National Institute for Public Health and the Environment, Centre for Nutrition Prevention and Health Services, Bilthoven, the Netherlands

**Keywords:** Colorectal cancer (CRC), Population based screening, Interviews, Decision-making, Informed decision-making, IDM

## Abstract

**Background:**

During the first year of the population based colorectal cancer (CRC) screening program on Curaçao, about 20% of invitees participated. This study explored the target population’s perceptions and awareness on CRC (screening), beliefs on the program provision, their preferences and information needs for informed decision-making.

**Methods:**

Semi-structured interviews with 23 individuals, who were not yet invited for CRC screening, were recorded, transcribed, coded and analyzed.

**Results:**

CRC (screening) was discussed in the context of personal health, where own responsibility and food were important. Cancer was perceived as an unpredictable disease that causes suffering and leads to death and was also associated with fear. Despite being aware of the program, most respondents were not familiar with the screening procedure. Provision of the screening program was regarded positively and as an opportunity to contribute to health improvement. This seemed related to the expressed trust in the Caribbean Prevention Center (program organizer). Respondents preferred to make independent decisions about CRC screening participation. A personal approach, visual aids and media were the preferred sources of information.

**Conclusion:**

The results of our interviews suggest that it may be beneficial to provide information on CRC screening in Curaçao within the context of personal health. While including sensitivity to fears and respect for the autonomy of the target population. Finally, electronic media maybe useful in supporting informed decision-making.

**Supplementary Information:**

The online version contains supplementary material available at 10.1186/s12889-023-16335-x.

## Background

Colorectal cancer (CRC) is the third most common type of cancer and the most common form of gastro-intestinal cancer globally [1]. Throughout the last two decades, CRC population screening programs have been responsible for the detection of substantial numbers of asymptomatic CRC cases worldwide [2]. This has resulted in timely treatment and global mortality reduction over time [3]. With this in mind, the Caribbean Prevention Center, or Fundashon Prevenshon (FP), as it’s known on Curaçao, launched the island’s first population screening program for CRC in 2020 [4]. This is a two-step fecal immunochemical test (FIT) based program. Those who test positive are invited to undergo a colonoscopy. Those who test negative are re-invited biennially. Inhabitants aged 50–75 years old are gradually invited by postal mail, according to their voting district. Because postal mail service is not always reliable, supportive radio and television media campaigns are provided. The campaign focuses on raising awareness of program availability. The FIT can be picked-up and dropped-off at multiple locations around the island.

Curaçao is an autonomous nation within the Kingdom of the Netherlands, located in the Caribbean. The population is multi-ethnic and multi-cultural, with most being Afro-Caribbean. There is also significant immigration from Latin America and Europe. More than 80% of the population speaks the local language of Papiamentu. Other languages make up the rest of the fraction, including Dutch, Spanish and English to name the most prevalent. Although the island is considered to have a high-income status for the Caribbean, the unemployment rate is relatively high at 19% [5, 6].

During the first year, approximately 20% of the population that was invited by postal mail completed a FIT [7]. For screening to be an effective public health strategy, adequate participation rates are needed [8, 9]. Decisions on screening participation should also be informed ones [10–13]. Informed decisions are defined as those that are based on relevant knowledge, consistent with the decision maker’s values and behaviorally implemented [14]. This means that the CRC screening target population should understand what CRC is, that the FIT is available, the pros and cons of screening and should be able to weigh these options and make a decision in line with their values.

The novel setting on Curaçao and low participation rate raise questions on how the target population possibly makes decisions on participating in CRC screening. Therefore, this study aims to gain insight into: (i) the target population’s awareness and perceptions of CRC (screening), (ii) their beliefs on the provision of the CRC screening program, (iii) their preferences regarding decision-making on CRC screening and (iv) what information they need to make an informed decision. The outcomes can be used to improve communication strategies and support informed decision-making in CRC screening in Curaçao and may also be relevant for other studies to build on.

## Methods

We conducted a qualitative study based on the grounded theory approach. Individual semi-structured interviews were carried out between September 2021 and February 2022. All interviews were scheduled and carried out by SB (MD, MSc), a female PhD-student, who is fluent in English, Dutch and Papiamentu.

### Sampling

To minimize the possibility of the shared opinions being influenced by personal experience with the screening program, those not yet invited for CRC screening (n = 37,380 out of n = 63,798) were identified in the FP database (Supplementary file 1). The “set.seed” function in R statistical programming was used to generate a random sample of 300, to ensure a large enough pool based on anticipated non-response and possible incomplete records. The FP database contains contact information. Of the 300 records, 164 did not include a phone number. Ultimately, phone calls were made to numbers belonging to 72 unique records. Snowball sampling was also used for additional interviews. During the initial phone call for invitation, the study aims and the interview procedure were explained and the status of program attendance was confirmed. If the individual agreed to be interviewed, an appointment was made at the FP main office, the respondent’s home or, if desired, by phone. Informed consent was obtained prior to each interview.

### Data collection

The interview topic-guide was developed by the research team (Supplementary file 2). The topic-guide consisted of five main domains and reflected the research goals of the study: CRC perception and awareness, perception and awareness of CRC-screening, CRC-screening provision, decision-making and information needs. All interviews lasted between 30 min to one hour and were recorded and transcribed verbatim in the original interview language. Subsequently, all non-English transcripts were translated into English. The English version of all transcripts were coded for analysis. During the interview period the research team met regularly to discuss the findings in order to ensure the process of reflexivity.

### Analysis

All transcripts were coded with MAXQDA software. First, transcripts were read and re-read by SB to become familiar with the data. Then a selection of transcripts were open coded by SB and MF, independently. Subsequently, themes were classified into code trees per research question. The final code trees were discussed and agreed upon by SB and MF. To ensure trustworthiness, each interview was then uniformly coded with the code trees by SB and TV, independently. No major discrepancies emerged from the comparison.

## Results

### Response and baseline characteristics of the research population

A total of 20 consecutive interviews resulted from the recruitment phone calls. An additional three interviews were carried out based on snowball sampling, resulting in a total of 23 interviews. The last five interviews did not introduce any new topics and therefore data saturation was observed.

In total 13 men and 10 women were interviewed (Table 1). The mean age of the respondents was 64.3 years, 65% had an intermediate level of education, 87% were of Caribbean decent, 52% were married and 48% employed at the time of the interviews. Most interviews (n = 17) were conducted in Papiamentu, the other interviews in English and Dutch.

**Table 1:** Baseline characteristics of the research population (n = 23)

Sex	
**Male, n (%)**	13 (56.5%)
**Mean age (SD)**	64.3 (6.9)
**Education level**	
**Low, n (%)**	3 (13%)
**Intermediate, n (%)**	15 (65%)
**High, n (%)**	5 (22%)
**Background**	
**Caribbean, n (%)**	20 (87% )
**European, n (%)**	2 (9%)
**Other, n (%)**	1 (4%)
**Marital status**	
**Married, n (%)**	12 (52%)
**Single, n (%)**	8 (35%)
**Divorced, n (%)**	3 (13%)
**Employment status**	
**Employed, n (%)**	11 (48%)
**Retired, n (%)**	11 (48%)
**Unemployed, n (%)**	1 (4%)
**Interview language**	
**Papiamentu, n (%)**	17 (74%)
**English, n (%)**	4 (17%)
**Dutch, n (%)**	2 (9%)

### Perceptions on health, cancer and CRC

#### Health, nutrition and health responsibility

Perceptions about cancer and cancer screening were frequently discussed in relation to the broader concepts of health and nutrition. Table 2 presents illustrative quotes. General ideas about disease and disease prevention were extrapolated to thoughts on cancer and cancer prevention by means of screening.

Food and nutrition were frequently discussed in the interviews. Nutrition choices were seen as an integral part of healthy living. The use of healthy food was considered to not only contribute to the preservation of health, but also as a potential source of healing. On the other hand, an unhealthy diet was regarded as a possible source of disease and a cause of cancer in particular (Table 2, Q01). The general view was that CRC specifically is a disease of the intestines, therefore a relationship with food and its consumption would be obvious (Table 2, Q02).

The respondents saw themselves as being more concerned with their general health than their peers. They described participation in health motivated activities, such as eating healthy or taking part in preventive practices such as screening as a personal responsibility. It was explicitly voiced that this feeling of responsibility is the core difference between those who are health conscious and those who are not. Meaning that those who are not health conscious also tend to act less responsible when it comes to their health.

The opinion that other people were not only inadequately health conscious, but also failed to prioritize their health, was prominent. This was even suggested as a possible reason why some peers would not attend the CRC screening program. Emphasizing this view, the example that others don’t go to the doctor when they should, was given (Table 2, Q03-4).

A group that was considered to be sufficiently health-conscious were the somewhat older people. Age is seen as a factor that allows for making good choices when it comes down to health (Table 2, Q05).

It was also speculated that a lack of health consciousness, could be related to not wanting to be confronted with anything negative, like being informed about having a disease (Table 2, Q06-07).

**Table 2:** Perceptions on health, cancer and CRC.

Theme	*Quote	*Respondent
**Food**	Q01. *“I eat quite healthy, and I see others who maybe don’t do anything or don’t pay attention to what they ingest in their body. And you see the difference right away, in how vital that person is or not.”*	R23, male, 70 years of age, intermediate education level
**Nutrition**	Q02. Interviewer: *“Do you have any ideas about what can cause colon cancer?”* Respondent: *“That is something that…well, I speculate is a way of eating. Let’s say, red meat…activates…well, I think sugar as well. But sugar is bad for people, so I believe that it can affect the intestines, I suppose…well I think in the big picture the way of eating.”*	R11, male, 63 years of age, high educational level
**Personal responsibility**	Q03. Interviewer: “*And why do you think that you see it a bit different from [others]?”* Respondent: *“They have no responsibility.”*	R06, female, 56 years of age, intermediate education level
**Health consciousness**	*Q04. “We don’t even want to go to the doctor for us to be conscious that we need to do a certain screening or a certain test …. So we are not conscious [of] those. ”*	R06, female, 56 years of age, intermediate education level
	*Q05. “Maybe the younger folks won’t worry so much…But the older folks, yes. Especially when you reach a certain age. I think it’s important.”*	R02, female, 64 years of age, intermediate education level
	*Q06. “There are people who don’t feel well and don’t go to the doctor. They don’t go because they don’t want to hear anything, they don’t want to hear.”*	R03, female, 68 years of age, intermediate education level
	*Q07. “They don’t want to hear anything bad. They don’t want to hear that I have it or that there is something or even when they hear it, because there’s that also, they keep it to themselves.”*	R06, female, 56 years of age, intermediate education level

*Quote: All quotes are numbered, with the letter *Q* and two digits.

*Respondent: for each quote the corresponding respondent is cited with the letter *R* and two digits

#### Beliefs about cancer: death, unpredictable, recurrence

Some respondents shared their personal experiences with cancer. For example, some of those who experienced cancer through relatives or friends, perceived cancer as fairly common. The disease was talked about like a general phenomenon that can show up at any point in anyone’s life (Table 3, Q01).

**Table 3:** Beliefs about cancer and CRC

Theme	*Quote	*Respondent
**Common**	Q01. Interviewer: *“You said you are accustomed to it, what do you mean?”* Respondent: *“Thus hearing about cancer. But you know, back in the days, they would whisper, they have cancer. Nowadays, no, I have cancer. It has become something a little more normal.”*	R05, male, 70 years of age, low education level
**Suffering**	*Q02. “Sometimes I feel treatment…you have to go through a lot. And I’m the type of person that doesn’t like to see others suffer.”*	R03, female, 68 years of age, intermediate education level
**Death**	Q03. Interviewer: *“When I say colon cancer, what comes to mind?”* Respondent: *“Uh, getting seriously ill. That is what comes to my mind and…relatives…aunts and grandmothers that have already died… because of cancer.”*	R17, male, 50 years of age, intermediate education level
	Q04. Interviewer: *“What is the first thing that you think about when I say colon cancer?”* Respondent: *“I’m going to die tomorrow.”* Interviewer: *“How come?”* Respondent: “*I don’t know. Uhm, well, because the cancer, from the moment you hear about cancer, you think you won’t be here much longer.”*	R21, male, 58 years of age, low education level
**Unknown, unpredictable**	*Q05. “Well, because uhm, the way they talk about cancer, so it’s not something that you feel, but it’s something that presents itself.”*	R18, female, 72 years of age, intermediate education level
**Unpredictable**	Q06. “…it can go different ways, right, as I understood.”	R11, male, 63 years of age, high education level
**Death, Recurrence**	*Q07. “Maybe because I have acquaintances who have died and stuff like that. Recently…I have a sister-in-law in Aruba and…well…she found out she has breast cancer. She had to do a lot of chemo and all that. And it still turned out that she needed to be operated on.”*	R03,female, 68 years of age intermediate education level
**Shame, Fear**	*Q08. “A lot of people are afraid, ashamed. Yes, as I said if it was back in the days, I would have been ashamed as well to say: “no, I have cancer”, you know? It makes you ashamed.”*	R05, male, 70 years of age, low education level
**Taboo**	*Q09. “If I look at how the Curaçaolean perceives certain illnesses that are taboos, that they hide certain illnesses, even COVID. If you get COVID, you hide that you got COVID or that you have cancer…you hide those….”*	R08, male, 72 years of age, high education level
**Taboo, Fear**	*Q10. “In Curaçao it is as if, I don’t know if it is taboo, fear or what it is but unfortunately there is a large group of the population who do not dare to take that step. They would rather, uhm, keep it a secret until unfortunately maybe a family member or someone else discovers something and then it’s too late.”*	R04, male, 54 years of age, intermediate education level
**Fear**	*Q11. “Some people [are] scared of different things. Maybe they do not want to know that they have something.”*	R16, female, 67 years of age, intermediate education level
	*Q12. “They don’t want to hear anything bad. They don’t want to hear that I have it or that there is something or even when they hear it, because theres that also, they keep it to themselves.”*	R06, female, 56 years of age, intermediate education level
	*Q13. “Sometimes it’s just the fear. The fear that a person has, [it] makes them….maybe they don’t feel well, but the fear to hear the truth or whatever or maybe they suspect…They will stay years without looking into the matter.”*	R03, ,female, 68 years of age intermediate education level
	*Q14. “I think fear of knowing, to hear something that is not good.”*	R13, female, 61 years of age, intermediate education level
	*Q15. “Let me tell you, cancer in general, the moment you hear cancer, you get goose bumps. So you get a lots and lots of shivers.”*	R07, female, 58 years of age, high education level
	*Q16. “I can- even though I tell you…really sincerely there are moments I have…I have like a…let’s say a fear you know…of the result. If I participate and the result says “Hey, you have cancer.” Damn…I’m very scared of that.”*	R08, male, 72 years of age, high education level
**Fear, Machismo**	*Q17. “I think men are more cowardly than women.”*	R18, female, 72 years of age, intermediate education level
**Machismo**	Q18. Respondent: *“They say always men are the ones that hide the sickness, so I always hear that. Men do not like to hear they are sick, they don’t tell people they are sick in any case.”* Interviewer: *“And what do you think that is rooted in?”* Respondent: “*I would have to say maybe it’s the mentality they have, they feel they are strong, they are always strong and they do not want to know they are weak. They don’t want to face weakness, weakness- sickness is a weakness. ‘I’m a man and so I’m not afraid of anything’.”*	R16, female, 67 years of age, intermediate education level
**Susceptibility**	*Q19. “My father passed away in October, but a few years ago he had a bleeding in his intestine. So I know. I experienced that process in the hospital, what that can be like for someone. The intestine isn’t something that you want to mess around with, it’s an important part of life… So I had my own interest in this research.”*	R17, male, 50 years of age, intermediate education level

*Quote: All quotes are numbered, with the letter *Q* and two digits

*Respondent: for each quote the corresponding respondent is cited with the letter *R* and two digits

The perceived consequences of the diagnosis were also discussed, namely a lot of emotional and physical suffering. Suffering would potentially arise primarily from the treatment following diagnosis such as surgery that is likely to result in pain (Table 3, Q02).

The association of cancer and death was often mentioned. Despite the knowledge that this doesn’t necessarily have to be the case if diagnosed in a timely fashion, death was cited as an immediate and automatic perceived consequence of having cancer. This association was sometimes based on personal experiences (Table 3, Q03-04).

The idea that cancer inevitably leads to death was highlighted by mentions of unpredictability and recurrence. The unknown aspects that surge within a person when cancer is mentioned were also discussed. From the possibility of it being present and you not knowing, because of the absence of symptoms or not knowing what steps will be taken to treat it all caused concern.

There was also the notion that the disease could always return even after treatment (Table 3, Q05-07).

#### Shame and taboo

In addition to the medical consequences of a cancer diagnosis, participants also mentioned the social implications of having cancer. For example, shame may emerge when others find out that an individual has been diagnosed with cancer (Table 3, Q08).

Mentioning cancer is also seen as taboo. Respondents stated that other people don’t want to talk about it, let alone face the possibility that they might have cancer by actively participating in screening (Table 3, Q09).

In addition, people may express their pity to a person with cancer. This was perceived as negative, as it is considered an expression of relief from those who do not have cancer (Table 3, Q10).

#### Fear

The interviews showed that people in some way feared cancer and its consequences. The fear most talked about was the fear of having to learn that one has cancer, the fear of being ill.

The impression that peers are afraid of getting cancer was also put forward as a reason why many people refrain from taking part in the CRC screening. Fear also seemed to be related to the aspect of the unknown, not knowing what the diagnosis of cancer means and what lies ahead. Finally, there was the fear of the possible social implications of being diagnosed with cancer. Although respondents mainly talked about others, some of them admitted to being afraid themselves. In these cases, it was mainly about the fear of being diagnosed with cancer (Table 3, Q11-16).

One group that was said to be especially fearsome were men. Respondents mentioned that men were less likely to be concerned about their health than women. That what is presented as bravery is usually a façade for the underlying cowardice (Table 3, Q17).

#### Machismo

Machismo was also listed as an important reason for local men not to attend the CRC screening program. The perception was that men see themselves as strong and somewhat invincible. Respondents indicated that men would rather not participate in anything that may reveal an illness. Sickness is perceived to be associated with weakness and vulnerability (Table 3, Q18).

#### CRC: causes and susceptibility

As previously mentioned, two perceived causes for cancer and in extrapolation CRC were eating habits and avoiding contact with the doctor when feeling unwell. Reasons that were explicitly mentioned to participate in CRC screening were: family history and older age. The respondents were aware that cancer in the family was a possible predictor for developing cancer and that aging is associated with a reduction in general health status. As mentioned above, some respondents perceived others to be more at risk to get cancer due to poor lifestyle choices and lack of health consciousness (Table 3, Q19).

### Beliefs on CRC screening

#### CRC screening as opportunity

As previously mentioned, health promoting behavior was considered very important amongst the respondents. Looking after one’s general physical health was seen as a task that should be taken seriously. Accordingly, the respondents were very positive about the CRC screening program, almost without considering any possible disadvantages.

The offer of a CRC screening test was mainly regarded as an opportunity to undergo a free health check or to gain knowledge of a still unknown malignancy through early detection, so that it could be treated in time (Table 4, Q01-02).

#### Awareness of CRC screening

Most respondents indicated that they were aware of the CRC screening program and its availability because some aspect of the media campaign reached them, such as a radio commercial or a television interview. However, they pointed out that they did not know what the process entailed or what they were supposed to do (the different steps in the program). Most respondents did not know that the FIT is the primary screening method and that the colonoscopy is only performed in case of a positive FIT. Furthermore, colonoscopy was also described as potentially painful (Table 4, Q03).

**Table 4:** Beliefs on CRC screening (provision)

Theme	*Quote	*Respondent
**Positive attitude**	Q01. Interviewer: “*And what do you think about the program that we have for screening that exists here in Curaçao?”* Respondent: “*Great, it is great. No, I find it very good, a good initiative. Something very good, indeed, indeed. Because, [I would not come on my own account].”*	R05, male, 70 years of age, low education level
**Opportunity**	*Q02. “About colon cancer, you say well, you know what happens is that, from the moment you talk about cancer it scares a lot of people. And because it scares you, when you get opportunities like this you have to take it. To do certain tests.”*	R15,male, 68 years of age, high education level
**Early detection**	*Q03. “If it’s detected early on it is possible that… if they detect it early on then they can help you.”*	R01, female, 74 years of age, intermediate education level
**Colonoscopy**	Q04. Respondent: *“Not that I like the idea of a whole pipe in your body…”* Interviewer: *“But the screening itself, do you know what it entails?”* Respondent: *“No, I don’t know.”* Interviewer: *“Okay, but you just told me about a pipe.”* *Respondent: “Yes they spoke to me; I think a light that looks into your system.”* Interviewer: *“Are you under the impression that that is the screening?”* Respondent: *“That is the screening, yes.”*	R22, male, 72 years of age, intermediate education level
**Trust**	Q05. Interviewer: “*And you think that it’s good that the FP offers the screening instead of let’s say an organization like the government or a general practitioner?”* Respondent: *Yes, yes, I think a foundation like this is much better. Because let me say for example if the hospital does it, it would be different. But if you place an organization focused on certain things, I think it will turn out well for everyone, because they have their routine, everything stays- I think it will run a lot better. For both sides, both for the patient and for you as well*.”	R11, male, 63 years of age, high education level

*Quote: All quotes are numbered, with the letter *Q* and two digits

*Respondent: for each quote the corresponding respondent is cited with the letter *R* and two digits

### Decision-making in CRC- screening

#### Decision not always deliberate or conscious

In general there seemed to be no apparent rationale behind the decision to participate in CRC screening. The respondents did not state any clear motivations or mentioned positive beliefs when asked whether or not they would participate (Table 5, Q01).

In some cases the presence of physical symptoms related to the intestines was perceived as a prompt to participate in CRC screening. This thought process indicated that the decision was not always completely unconscious.

#### Autonomy

Having autonomy was described as paramount to several respondents, as individuals ultimately want to make their own choices. The respondents clearly indicated that they really did not want to be influenced by others when making a decision related to their physical health, because participating should be a personal choice.

Although the ultimate choice was made alone, it was noted that the experiences and opinions of family members and peers were also taken into consideration. Consulting with family members and peers was seen as valid when weighing options (Table 5, Q02-05).

**Table 5:** Decision-making

Theme	*Quote	*Respondent
**Undeliberate**	*Q01. “I have nothing to do, I have nothing to lose.”*	R05, male, 70 years of age, low education level
**Autonomy**	*Q02. “It’s my body, it’s my body. I have to decide, I can’t ask my spouse.”*	R05, male, 70 years of age, low education level
	*Q03. “…I have been living alone, I mean there are certain things you have to consult but not all. Things like this you have to do for your health, you can’t wait for people to tell you, and you have to take matters in your hands.”*	R16, female, 67 years of age, intermediate education level
	*Q04. “… I make my own decisions, because I live alone and my child lives in the Netherlands, so I make my own decisions.”*	R20, female, 59 years of age, intermediate education level
**Consulting others**	Q05. Interviewer: *“Do the opinions of others play a role when making a decision to participate or not?”* Respondent: *“Yes. I think so. Maybe if, depends on what you hear other people say, the more opinions you hear or more people you hear who are positive, I think that it then helps more people to go.”*	R13, female, 61 years of age, intermediate education level

*Quote: All quotes are numbered, with the letter *Q* and two digits

*Respondent: for each quote the corresponding respondent is cited with the letter *R* and two digits

### Information preferences

#### Means for information transfer

The use of media (television, radio, social media and applications) were mentioned as the preferred way to receive information and also seen as an effective method to inform others.

Respondents also mentioned that a multi-facetted approach, repetition and representation can be important tools when attempting to inform the public about CRC screening.

A personal approach was suggested as support to media coverage. For example a conversation with a FP employee, a phone call or text message. The use of lectures and visual tools were also suggested to be potentially helpful. Lastly, it was highlighted that information should be short, simple and phased (Table 6, Q01-03).

#### Content information

Most respondents indicated that providing the information in plain and intelligible language was of the utmost importance. This mainly concerns background information about what CRC entails, CRC epidemiology, risk factors for CRC and symptoms of the disease. In addition, the respondents indicated that an explanation about the screening process would also be useful. For example by making it clear that the first step of the CRC-screening program is the FIT and not the colonoscopy (Table 6, Q04).

**Table 6:** Information preferences

Theme	*Quote	*Respondent
**Media**	*Q01. “Promote the program a little more….put a little more programs…maybe on the radio, on the television, whatever in the media.”*	R02, female, 64 years of age, intermediate education level
**Multi-facetted**	*Q02. “You have a campaign going on, and I listened to the promo on the radio. It is…it is good, it’s effective. You should be more focused on prevention of how someone can prevent the ruin of their life environment both mentally, spiritually and physically. It is a priority, I believe more in a holistic form. Human beings, you have to accept them…in one form…you know, so in their totality of life, so life as a totality of savor.”*	R08, male, 72 years of age, high education level
**Information format**	*Q03. “It doesn’t have to be detailed ….short and sweet….Tell them exactly what the test does. So, if you explain to them that it is not complicated, I think that they will at least think about.”*	R04, male, 54 years of age, intermediate education level
**Information content**	*Q04.* ***“*** *I think the type of promotion that you are doing now, it has to become a different type of promotion. Where you show the instrument that you use or what the test entails precisely, to remove the fear or taboo.”*	R18, female, 72 years of age, intermediate education level

*Quote: All quotes are numbered, with the letter *Q* and two digits

*Respondent: for each quote the corresponding respondent is cited with the letter *R* and two digits

## Discussion

Our interviews revealed that perceptions of cancer, CRC and CRC screening are embedded in the larger context of personal health and prevention, where food plays an important role. Health maintenance is considered as a personal responsibility. Fear of being sick, the potential social implications of cancer (shame and taboo) and machismo among men seem to be important barriers to screening. Beliefs on the provision of CRC screening are positive and related to trust in the FP. Autonomy in decision-making is highly valued, whereby advice from others is appreciated. The need for information mainly relates to lack of knowledge on screening procedure. A personal approach, visual aids and (social) media were reported to be the preferred means of receiving information.

The perceived connection between cancer, CRC (screening) and personal health maintenance is interesting. A systematic review on barriers and facilitators of CRC screening reported that the desire to stay healthy and have peace of mind are motivators for screening [15]. Our respondents associated the perceived lack of responsibility and health consciousness of their peers with not participating in CRC screening. Previous qualitative studies also found that participation in cancer screening is regarded as part of healthy living [15, 16].

Food was seen as a true determinant of overall health, both capable of healing and the contrary, illness induction. Cultural beliefs surrounding food may play a role. Interviews about perceptions on CRC (screening) with 147 Puerto Ricans and Dominicans living in Rhode Island also revealed that the thought that consuming “bad food” potentially caused CRC was common [17]. In a smaller study about diabetes, treatment with people from a rural community in St. Vincent the healing power of traditional food was identified as an important cultural belief [18]. A qualitative study conducted amongst first generation non-Western immigrants in the Netherlands also found that all interviewed ethnic minority groups thought that there was an association between “non-natural” foods and the development of CRC [19].

The fear of being diagnosed with cancer and of the unknown mentioned during the interviews seem to be primarily caused by negative beliefs on the consequences of cancer, such as death and the unpredictability when it comes to treatment. A systematic review and meta-synthesis on CRC screening participation described such negative beliefs as barriers to screening [15]. In a large British review of qualitative literature on decision-making in screening, fear of having cancer was cited as a common barrier to screening [20]. Interestingly, fear was also found to be a facilitator, as cancer could remain uncovered if one were to not participate [20, 21]. The latter did not come up in our study, but when considering that respondents did admit to being afraid of the diagnosis themselves, maybe the positive attitude mentioned towards screening in combination with the negative ideas about cancer can be explained by this phenomenon.

Taboo and shame were brought up in relation to how others may perceive a cancer diagnosis. This points to the anticipation of social stigma. Perceived stigma is a widely discussed topic in cancer literature, and has been related to depression and decreased quality of life [22, 23]. Concern for what others may think has been found in other studies focusing on the Afro-Caribbean population. This sometimes results in the belief that the diagnosis should not be shared with others [24–26]. Our respondents did not explicitly express this. They only mentioned that the anticipated social consequences were a downside of a possible diagnosis. Social taboo and stigma after breast cancer diagnosis were indeed identified as barriers to screening in a review about breast cancer in the Caribbean [27].

In our study, machismo seemed to be a barrier to screening for men. This is in line with previous findings based on interviews with Mexican Americans [28]. The potential role of machismo is even more important when considering that men are less likely than women to participate in the FP CRC screening program, while they account for more than half of the detected CRC cases [7]. It has been previously reported in a systematic review and meta-analysis focusing on screening participation of men that the colonoscopy may be the reason for apprehension [29]. The colonoscopy related insertion disgust and fear of pain have been found to be associated to screening non-participation [30–34]. However, when the hypothesis of the degree to which upholding masculine ideals (i.e., self-reliance, risk-taking, heterosexual self-presentation, and primacy of work) was associated with colonoscopy adherence amongst veterans in the United States was tested, they found that greater endorsement of masculine ideals did not predict screening behavior [35].

As for the offer of CRC screening, the sentiment was generally positive and participation was seen as an opportunity to improve health. A positive view on cancer screening has also been observed in other studies that focused on the perceptions of cancer screening target populations. Mainly because early detection was considered to offer a better chance of recovery and would usually require less treatment [36–38]. Despite the positivity, even countries considered to have a high uptake, such as the Netherlands (68%) do not manage to get everyone to participate [2]. It is likely that those who are more inclined to participate in research about screening, have a more positive opinion on the subject, which would explain this finding emerging so often.

The positive beliefs also seem related to the trust that respondents had in the FP. The FP has history with the community. It is the only screening organization on the island. The organization has provided free screening for breast and cervical cancer since 2010 and 2014, respectively [7]. The trust may arise from the perception that the CRC screening program is another contribution from the FP to the health of the population. A previous study showed that participation in other screening programs is associated with a positive attitude towards a new cancer screening program [39].

However, trust in health care organizations is not self-evident, especially not in Caribbean populations. An evaluation of the health care system performance on vector borne diseases in Curaçao found that lack of coordination between the government and non-governmental organizations had negative effects [40]. This may possibly generate mistrust in the overall health care system. A review on the mental health of Afro-Caribbean people highlighted that there are “circles of fear” in this community that stop them from seeking help and stop health care professionals from properly engaging them [41]. Unconscious mistrust of those in perceived positions of authority, especially when it comes to making decisions on the body maybe related to the island’s history [41]. Curaçao gained autonomy within the Kingdom of the Netherlands in 2010. Like the entire Caribbean, the common history of slavery and colonialism cannot be ignored [41–43]. This is also discussed in a review looking at African-American CRC screening uptake in relation to medical mistrust. They concluded that higher levels of mistrust were associated with lower CRC screening attendance [44].

The expressed desire to make decisions independently may also be related to the past colonialism. This manifests itself in a need for control over one’s own body and freedom of choice in making decisions about health. The need for autonomy may also be related to fears about cancer, its treatment and cancer detection discussed above.

To make autonomous informed decisions certain skills are necessary. Namely, the ability to find, understand and apply information. These are called health literacy skills [45, 46]. To the knowledge of the authors, no data is currently available on health literacy on the island. Participants in our interviews seemed to have difficulty understanding the actual screening procedure. For example, they did not know that colonoscopy is only advised after a positive FIT. Colonoscopy was perceived as the first and only step, which may be painful. They also mentioned having symptoms as a motivator to screen. In the case of symptoms, a colonoscopy is more likely to be recommended. Aversion to colonoscopy is also known to sometimes be a reason to be apprehensive about CRC screening, although our respondents did not mention this explicitly [15, 31, 32, 47].

In line with our findings, a Jamaican survey study exploring knowledge and attitudes on the concept of CRC screening of 324 people found that only 14% of the subjects had not heard of CRC screening, but 69% stated that they did not know enough about it, with 32% not being familiar with any screening test [48]. In a Dutch study where knowledge of CRC screening program procedure was tested, 64% of the study population answered the questions about the program procedure accurately [49]. Limited knowledge has been found to be a barrier to screening, therefor providing sufficient information is important [15, 50]. Considering the growing importance of (social) media, it was not unexpected that the respondents would prefer and recommend it as a means of communication for a cancer screening program. At present, over 90% of the inhabitants of Curaçao use a mobile phone and about 70% have access to the internet [51]. For cancer patients, the preferred information source remains their physicians [52, 53]. Also, a known predictor for CRC screening is discussing it with a doctor [30, 54]. However, in a 2020 scoping review about the use of social media in cancer screening campaigns, the authors found that it may achieve better engagement and sometimes even translate to screening uptake [55].

### Strengths and limitations

A distinct strength of our qualitative semi-structured interviews is the unfiltered insight into the thought process of these members of the target population. Additionally, similar numbers of men and women were interviewed, coming from various social backgrounds, thereby offering variability within the group.

Because interviews were only conducted with people who explicitly consented after being informed of the purpose, the study results may be biased in favor of those who are more health-conscious, have a more positive attitude towards CRC screening and trust in the FP than those who did not consent. In addition to a positive attitude, it seemed that our respondents did not perceive any downsides to CRC screening. However, to reach an informed decision the pros and cons should be weighed. Furthermore, an interview with a medical doctor (SB) may have been a source of response bias where participants gave socially acceptable answers due to who was asking the questions, while knowing that a scientific research paper would be written. Respondents were encouraged to speak freely. They were informed that the goal was to explore their perceptions, awareness and possible insights on why the target population would (not) participate in CRC screening.

Some aspects of our study were dually strengths and weaknesses. The fact that we interviewed individuals who had not yet participated in screening meant that some of the answers were hypothetical, which is a possible limitation. Simultaneously, it offered the opportunity to hear perspectives unbiased by experience with the program, which is also a strength. Respondents often voiced their observations through a generalist lens, commenting primarily on the behaviors and perceived thought processes of others within society. The strength is the insight on how they perceived themselves in relation to others, as it suggests that they may feel disconnected from their peers. Overwhelmingly, the opinions of respondents on others were not positive, suggesting that respondents saw themselves as somewhat better. The limitation in this form of communication is that it may leave room for interpretation. The opinion of someone else could purely be a reflection of how they perceive others or their own disguised opinion about an issue. The interviewer usually tackled this by asking directly what made the interviewee different from others. Furthermore, in our results we limited the inclusion of respondent opinions on others. We did however, include these perceptions when relevant.

### Implications for practice

Personal health maintenance in relation to screening can be covered in information materials, while addressing possible fears. For example by highlighting favorable survival outcomes when CRC is detected early [2]. It is important to include that the FIT is the first step in CRC screening, and that it’s non-invasive and painless. Taking the desire for autonomy in decision-making into account, we recommend that information materials be clearly written in the language spoken and read by the target population. Adding (social) media to support the postal mail is likely to be the most effective way of reaching potential screening participants. The effect of new campaigns should also be evaluated, this was often missing in the results of previous studies [55]. In addition, the suggested personal approach is an opportunity to introduce creativity into the program and to acknowledge certain unique features of the culture. An example is the implementation of salon or barber shop-based interventions as conceptualized in the African American setting [56, 57].

### Implications for further research

The current study has provided useful insights, but has also made clear that there are still areas to be explored. Further insight is needed on how masculine ideals may influence CRC screening participation. It would also be interesting to investigate how health literacy plays a role in CRC screening on Curaçao. Additionally, quantitative research to investigate how and to what extent the identified factors play a role in decision-making in CRC screening maybe useful.

The insights gained about decision-making in our study could also be beneficial to other Caribbean islands, where they have not yet implemented organized CRC screening. The population of the Caribbean totals about 44 million [58]. In addition, the findings may also be informative for other settings, such as Caribbean migrant communities in Europe or African nations.

## Conclusion

To effectively inform the target population and assist informed decision-making, CRC screening should be framed within the broader perspective of health on Curaçao with sensitivity to possible fears and the expressed preference for independence in decision-making. Social media could be applied to disseminate targeted information about the CRC screening program and support invitees in their decision-making process.

## Electronic supplementary material

Below is the link to the electronic supplementary material.

Supplementary Material 1

Supplementary Material 2

Supplementary Material 3

Supplementary Material 4

Supplementary Material 5

## Data Availability

The data on which the conclusions of this study are based are available from the corresponding author upon reasonable request.

## List of abbreviations

CRC: Colorectal cancer
FIT: Fecal immunochemical test
FP: Caribbean Prevention Center, also known as Fundashon Prevenshon

## References

[1] Cancer. https://www.who.int/news-room/fact-sheets/detail/cancer. Accessed 6 Sep 2022.

[2] Navarro M, Nicolas A, Ferrandez A, Lanas A (2017). Colorectal cancer population screening programs worldwide in 2016: an update. World J Gastroenterol.

[3] Rawla P, Sunkara T, Barsouk A (2019). Epidemiology of colorectal cancer: incidence, mortality, survival, and risk factors. Gastroenterol Review/PrzeglÄ d Gastroenterologiczny.

[4] Bowel cancer screening program. https://www.fundashonprevenshon.com/bowel-cancer-screening. Accessed 24 Aug 2021.

[5] Curaçao. https://www.paho.org/en/curacao. Accessed 29 Nov 2022.

[6] Labour. https://www.cbs.cw/labour. Accessed 29 Nov 2022.

[7] Fundashon Prevenshon Annual Report. 2020. https://www.fundashonprevenshon.com/annual-report.

[8] Symonds EL, Pedersen S, Cole SR, Massolino J, Byrne D, Guy J (2015). Improving participation in Colorectal Cancer Screening: a Randomised Controlled Trial of Sequential offers of Faecal then blood based non-invasive tests. Asian Pac J Cancer Prev.

[9] de Bekker-Grob EW, Donkers B, Veldwijk J, Jonker MF, Buis S, Huisman J (2020). What factors influence non-participation most in Colorectal Cancer Screening? A Discrete Choice Experiment. Patient - Patient-Centered Outcomes Res.

[10] Andermann A, Blancquaert I, Beauchamp S, Déry V (2008). Revisiting Wilson and Jungner in the genomic age: a review of screening criteria over the past 40 years. Bull World Health Organ.

[11] Wilson JMG, Jungner G. World Health Organization: principles and practice of screening for disease. World Health Organization. 1968. https://apps.who.int/iris/handle/10665/37650.

[12] Marteau TM, Dormandy E, Michie S (2001). A measure of informed choice. Health Expect.

[13] Rimer BK, Briss PA, Zeller PK, Chan ECY, Woolf S (2004). Informed decision making: what is its role in cancer screening?. Cancer.

[14] O’Conner A, O’Brien-Pallas L. Decisional conflict In: Mcfarlan G, Mcfarlane E, editors. *Nursing Diagnosis and Intervention*. Toronto, Canada:Mosby; 1989. 486–96.

[15] Honein-AbouHaidar GN, Kastner M, Vuong V (2016). Systematic review and Meta-study synthesis of qualitative studies evaluating facilitators and barriers to participation in Colorectal Cancer Screening. Cancer Epidemiol Prev Biomarkers.

[16] Jilcott Pitts SB, Lea CS, May CL, Stowe C, Hamill DJ, Walker KT (2013). Fault-line of an Earthquake”: a qualitative examination of barriers and facilitators to Colorectal Cancer Screening in Rural, Eastern North Carolina. J Rural Heal.

[17] Goldman RE, Diaz JA, Kim I (2009). Perspectives of colorectal cancer risk and screening among Dominicans and Puerto ricans: Stigma and misperceptions. Qual Health Res.

[18] Moss MC, McDowell JRS (2005). Rural Vincentians’ (Caribbean) beliefs about the usage of non-prescribable medicines for treating type 2 diabetes. Diabet Med.

[19] Woudstra AJ, Dekker E, Essink-Bot ML, Suurmond J (2016). Knowledge, attitudes and beliefs regarding colorectal cancer screening among ethnic minority groups in the Netherlands - a qualitative study. Health Expect.

[20] Young B, Bedford L, Kendrick D, Vedhara K, Robertson JFR, Das Nair R (2018). Factors influencing the decision to attend screening for cancer in the UK: a meta-ethnography of qualitative research. J Public Health (Oxf).

[21] Dressler J, Johnsen AT, Madsen LJ, Rasmussen M, Jorgensen LN (2021). Factors affecting patient adherence to publicly funded colorectal cancer screening programmes: a systematic review. Public Health.

[22] Ernst J, Mehnert A, Dietz A, Hornemann B, Esser P (2017). Perceived stigmatization and its impact on quality of life - results from a large register-based study including breast, colon, prostate and lung cancer patients. BMC Cancer.

[23] Chambers SK, Dunn J, Occhipinti S, Hughes S, Baade P, Sinclair S (2012). A systematic review of the impact of stigma and nihilism on lung cancer outcomes. BMC Cancer.

[24] Parveen S, Peltier C, Oyebode JR (2017). Perceptions of dementia and use of services in minority ethnic communities: a scoping exercise. Heal Soc Care Community.

[25] Shafiq S, Parveen S, Oyebode JR (2021). How people of african caribbean or irish ethnicity cope with long-term health conditions in UK community settings: a systematic review of qualitative, quantitative and mixed method studies. Heal Soc Care Community.

[26] Sisley EJ, Hutton JM, Louise Goodbody C, Brown JSL (2011). An interpretative phenomenological analysis of african caribbean women’s experiences and management of emotional distress. Heal Soc Care Community.

[27] Samaroo K, Hosein A, Olivier LK, Ali J (2021). Breast Cancer in the Caribbean. Cureus.

[28] Hunter JB, Fernandez ML, Lacy-Martinez CR, Dunne-Sosa AM, Coe MK (2007). Male Preventive Health Behaviors: perceptions from men, women, and clinical staff along the U.S.—Mexico Border. Am J Men’s Health.

[29] Teo CH, Ng CJ, Booth A, White A (2016). Barriers and facilitators to health screening in men: a systematic review. Soc Sci Med.

[30] Reynolds LM, Bissett IP, Consedine NS (2018). Emotional predictors of bowel screening: the avoidance-promoting role of fear, embarrassment, and disgust. BMC Cancer.

[31] Kerrison RS, Travis E, Dobson C, Whitaker KL, Rees CJ, Duffy SW (2022). Barriers and facilitators to colonoscopy following fecal immunochemical test screening for colorectal cancer: a key informant interview study. Patient Educ Couns.

[32] Alduraywish SA, Altamimi LA, Almajed AA, Kokandi BA, Alqahtani RS, Alghaihb SG (2020). Barriers of colorectal cancer screening test among adults in the Saudi Population: a cross-sectional study. Prev Med Reports.

[33] Chambers JA, Callander AS, Grangeret R, O’Carroll RE (2016). Attitudes towards the Faecal Occult Blood Test (FOBT) versus the Faecal Immunochemical Test (FIT) for colorectal cancer screening: perceived ease of completion and disgust. BMC Cancer.

[34] Scaglioni G, Guidetti M, Cavazza N (2021). The role of disgust as an emotional barrier to colorectal cancer screening participation: a systematic review and meta-analysis. Psychol Health.

[35] Christy SM, Mosher CE, Rawl SM, Haggstrom DA (2017). Masculinity beliefs and colorectal Cancer screening in male veterans. Psychol Men Masc.

[36] Waller J, Osborne K, Wardle J (2015). Enthusiasm for cancer screening in Great Britain: a general population survey. Br J Cancer.

[37] Hall NJ, Rubin GP, Dobson C, Weller D, Wardle J, Ritchie M (2015). Attitudes and beliefs of non-participants in a population‐based screening programme for colorectal cancer. Health Expect.

[38] Woudstra AJ. Informed decision making in colorectal cancer screening: Equal opportunities for disadvantaged groups [Thesis, fully internal, Universiteit van Amsterdam].2020. https://hdl.handle.net/11245.1/2ee17aac-2e28-4d33-b132-93123ed17c47.

[39] Cullati S, Charvet-Bérard AI, Perneger TV (2009). Cancer screening in a middle-aged general population: factors associated with practices and attitudes. BMC Public Health.

[40] Mulderij-Jansen V, Gerstenbluth I, Duits A, Tami A, Bailey A (2021). Evaluating and strengthening the health system of CuraÒ«ao to improve its performance for future outbreaks of vector-borne diseases. Parasit Vectors.

[41] Keating F, Robertson D. Breaking the circles of fear. OPEN MIND Published online 2002:16–7. Accessed September 29, 2022. www.scmh.org.uk.

[42] Sastre F, Rojas P, Cyrus E, De La Rosa M, Khoury AH (2014). Improving the health status of caribbean people: recommendations from the triangulating on Health Equity summit. Glob Health Promot.

[43] Serbín A (1994). Towards an Association of Caribbean States: raising some awkward questions. J Inter Am Stud World Aff.

[44] Adams LB, Richmond J, Corbie-Smith G, Powell W (2017). Medical Mistrust and Colorectal Cancer Screening among African Americans. J Community Health.

[45] Woudstra AJ, Timmermans DRM, Uiters E, Dekker E, Smets EMA, Fransen MP (2018). Health literacy skills for informed decision making in colorectal cancer screening: perceptions of screening invitees and experts. Health Expect.

[46] Van Der Heide I, Uiters E, Jantine Schuit A, Rademakers J, Fransen M (2015). Health literacy and informed decision making regarding colorectal cancer screening: a systematic review. Eur J Public Health.

[47] Van Dam L, Korfage IJ, Kuipers EJ, Hol L, van Roon A, Reijerink J (2013). What influences the decision to participate in colorectal cancer screening with faecal occult blood testing and sigmoidoscopy?. Eur J Cancer.

[48] Lee MG, Brown EF, Mills MO, Walters CA (2017). Colon cancer screening: knowledge and attitudes in a jamaican Population and Physicians. World J Res Rev.

[49] Essink-Bot ML, Dekker E, Timmermans DRM, Uiters E, Fransen MP (2016). Knowledge and informed decision-making about Population-Based Colorectal Cancer Screening participation in groups with low and adequate health literacy. Gastroenterol Res Pract.

[50] van den Berg M, Timmermans DR, ten Kate LP, van Vugt J, van der Wal G (2006). Informed decision making in the context of prenatal screening. Patient Educ Couns.

[51] People and Society. | Curacao Data CBS. https://curacaodata.cbs.cw/people-and-society. Accessed September 29, 2022.

[52] Chua GP, Tan HK, Gandhi M (2018). Information sources and online information seeking behaviours of cancer patients in Singapore. Ecancermedicalscience.

[53] Mekuria AB, Erku DA, Belachew SA (2016). Preferred information sources and needs of cancer patients on disease symptoms and management: a cross-sectional study. Patient Prefer Adherence.

[54] Goodwin BC, Ireland MJ, March S, Myers L, Crawford-Williams F, Chambers SK (2019). Strategies for increasing participation in mail-out colorectal cancer screening programs: a systematic review and meta-analysis. Syst Rev.

[55] Sinky TH, Faith J, Lindly O, Thorburn S (2018). Cancer Fatalism and Preferred sources of Cancer Information: an Assessment using 2012 HINTS Data. Cancer Educ.

[56] Rogers CR, Okuyemi K, Paskett ED, Thorpe RJ jr, Rogers TN, Hung M, et al. Study protocol for developing #CuttingCRC: a barbershop-based trial on masculinity barriers to care and colorectal cancer screening uptake among african-american men using an exploratory sequential mixed-methods design. BMJ Open. 2019;9(7):e030000. 10.1136/BMJOPEN-2019-030000. 10.1136/bmjopen-2019-030000 PMC6661686 31345981

[57] Floyd TD, DuHamel KN, Rao J, Shuk E, Jandorf L (2017). Acceptability of a salon-based intervention to promote Colonoscopy Screening among African American Women. Health Educ Behav.

[58] Population. total – Latin American & Caribbean. https://data.worldbank.org/indicator/SP.POP.TOTL?locations=ZJ. Accessed 20 January 2023.

