# Supplementary material for: Decision-making on colorectal cancer screening in Curaçao - interviews with the target population
Source: BMC Public Health. 2023 Jul 27;23:1437. doi: 10.1186/s12889-023-16335-x (PMC10373279; doi:10.1186/s12889-023-16335-x)
Supplement: Supplementary file 1 — Supplementary Material 1 [file 12889_2023_16335_MOESM1_ESM.pdf]

Supplementary file 1
Flowchart: Sampling research population

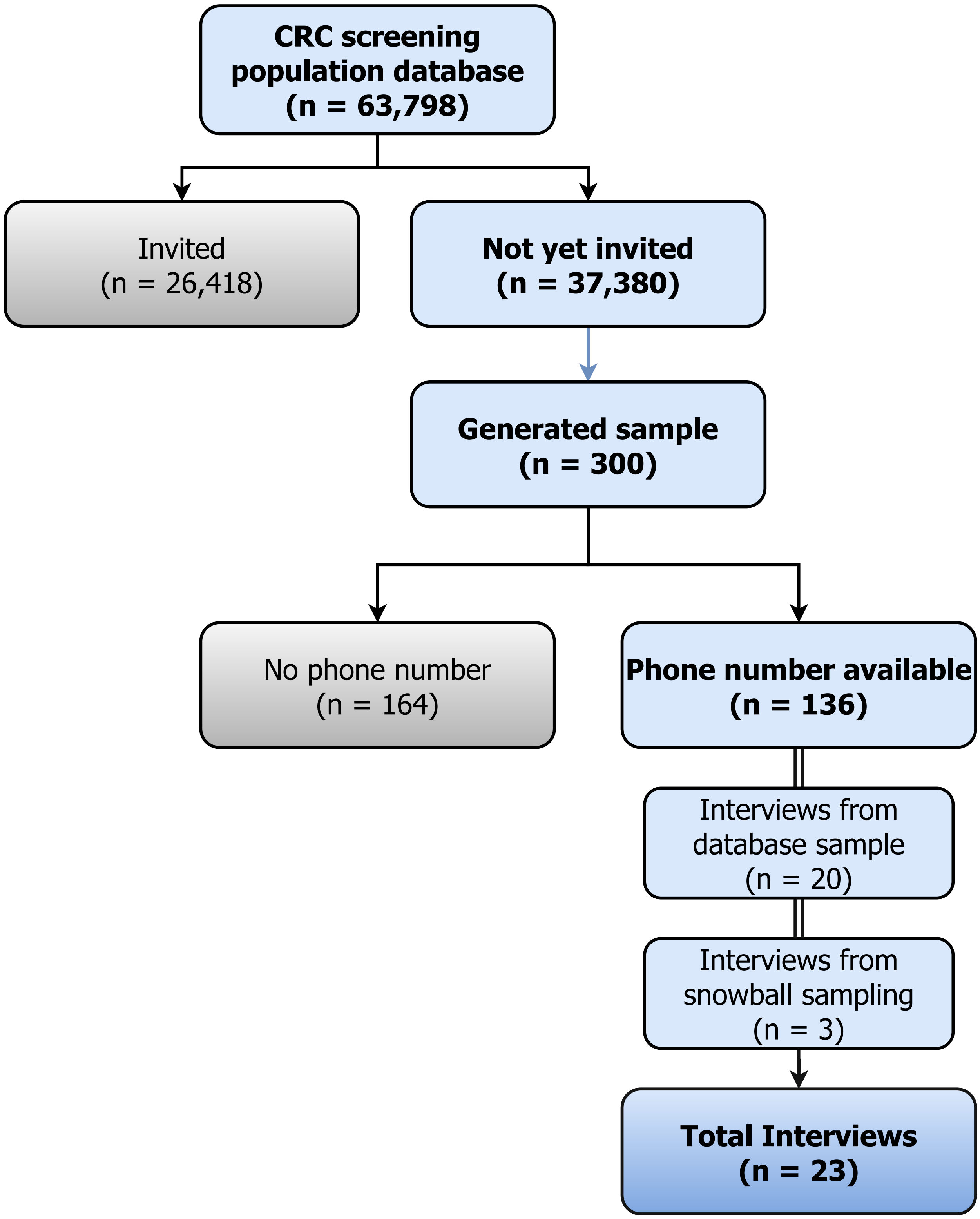

Selection of the study population from the screening organization database: First, those not yet invited for CRC screening were identified (n=37,380). Second, 300 records were randomly selected in R statistical programming. Only 136 records contained a telephone number. 72 phone calls were made. This resulted in 20 interviews. An additional 3 interviews were conducted based on snowball sampling.
