# Supplementary material for: Decision-making on colorectal cancer screening in Curaçao - interviews with the target population
Source: BMC Public Health. 2023 Jul 27;23:1437. doi: 10.1186/s12889-023-16335-x (PMC10373279; doi:10.1186/s12889-023-16335-x)
Supplement: Supplementary file 2 — Supplementary Material 2 [file 12889_2023_16335_MOESM2_ESM.docx]

# Introduction

- Good day, my name is []
- I am a PhD student at []. I am currently doing research on colorectal cancer (CRC) screening.
- Today I would like to interview you about CRC screening.
- The interview will last a maximum of 1 hour.

# Research Goals and Questions

The aim of this study is to better understand how the population makes decisions on CRC screening. This knowledge can be used to adapt the CRC screening program invitations and information materials to effectively invite and inform the target population to support informed decision-making.

The specific research questions are:

- What are the target population’s awareness and perceptions on CRC and CRC screening on Curaçao?
- What are their beliefs towards the provision of CRC screening?
- What preferences do they have regarding decision-making on CRC screening?
- What information do they need to decide whether or not to participate in CRC screening?

# Disclaimer

- All information gathered is confidential and will be used for research purposes only.
- The interviewee may stop the interview at any time for any reason.
- Interview will be recorded.

## Information letter and informed consent

- Read information letter and informed consent aloud with interviewee.
- Sign informed consent and ask interview to sign as well.

## Recording

- Let interviewee know when the recorder is going to be turned on.
- Start recording.
- State date, name of interviewer, respondent number.
- Check recording quality.

# Topic guide layout

This topic guide has been divided into domains to reflect the research questions.

# Interview questions in Domains

1. **Domain: CRC perception and awareness**

- When I say CRC what do you think? What comes to mind?
  - Where does that stem from? (Can you elaborate?)
  - What do you think causes CRC?
  - Who do you think usually gets CRC? Do you think you can get it?
  - Have you had any experience with CRC in your own life (family, friends, and acquaintance)? How did that make you feel? How did that affect your opinion on CRC?
- What do you think the ideas of the community about CRC are?
  - Where do you think that comes from? (Can you explain?) What is your opinion on this?

1. **Domain: CRC screening perception and awareness**

- What are your ideas on CRC screening?
  - Have you ever participated in any other population-screening program?
  - Would it be a good thing for you to do? Why?
  - What would motivate you attend? What would stop you from attending (own ideas, ideas from others, religion, transportation)?
  - Do you know how it works? (If it comes up: do you know what a FIT is? a colonoscopy?)
  - What role would you say the opinions of others play?
  - How does [any of the above] make you feel about attending the CRC screening program? Why? (Would anything change that?)

1. **Domain: Provision of CRC screening on Curaçao**

- Have you heard about the CRC screening program on Curaçao? What do you think about it?
- What do you think about the program being available on the island? (Is it a good idea? Is it helpful or useful?) Why? Do you have any expectations for the program?
- What do you think the community thinks about the CRC screening program being available on Curaçao?
- What organization do you think should be offering CRC screening to the public? (FP, GP, Government?)
- What do you think about attendance being voluntary? Do you think somebody should give you advice on whether or not to attend?

1. **Domain: Information needs**

- If you were a program organizer, how would you invite the public? What methods (letter, tv, bus etc.) would you use? (Why?)
- What do you think we should do? (Why?)
- What kind of information would help you make a choice?
- What is your opinion on observed information materials? Do they portray the message?
- If you could change something about the available information, what would you change? Why?

1. **Domain: Decision-making**
   - Do you need additional support to make a choice (family, friends, GP, FP)?
   - Do you feel you could make a choice at this moment?
     - If yes: what makes it possible for you to make a choice (family, friends, hearing about it)? Are you doubtful? (If so, why?) Is there anything that can change your mind? (Please elaborate)
     - If not: can you explain why? Is there anything that can help you make a choice? Is there anything standing the way of you making a choice? (Please elaborate)

**Domain: Participant information**

- Where were you born?
- Where were your parents born?
- How do identify culturally?
- What language do you speak at home?
- Do you currently have paid work?
- What is the highest level of education you have completed?
- What is your marital status?
- Do you have any children?

# Closing

- Was this interview what you expected?
- Was there anything you would like to discuss further? A topic you would like to re-visit?
- If appropriate: Offer explanation on screening, CRC or CRC screening.
- Thank interviewee for their time.
