# Supplementary figures and images for: Decision-making on colorectal cancer screening in Curaçao - interviews with the target population
Source: BMC Public Health. 2023 Jul 27;23:1437. doi: 10.1186/s12889-023-16335-x (PMC10373279; doi:10.1186/s12889-023-16335-x)

### Supplementary file 3

#### Interview themes about perceptions of cancer, CRC and CRC screening

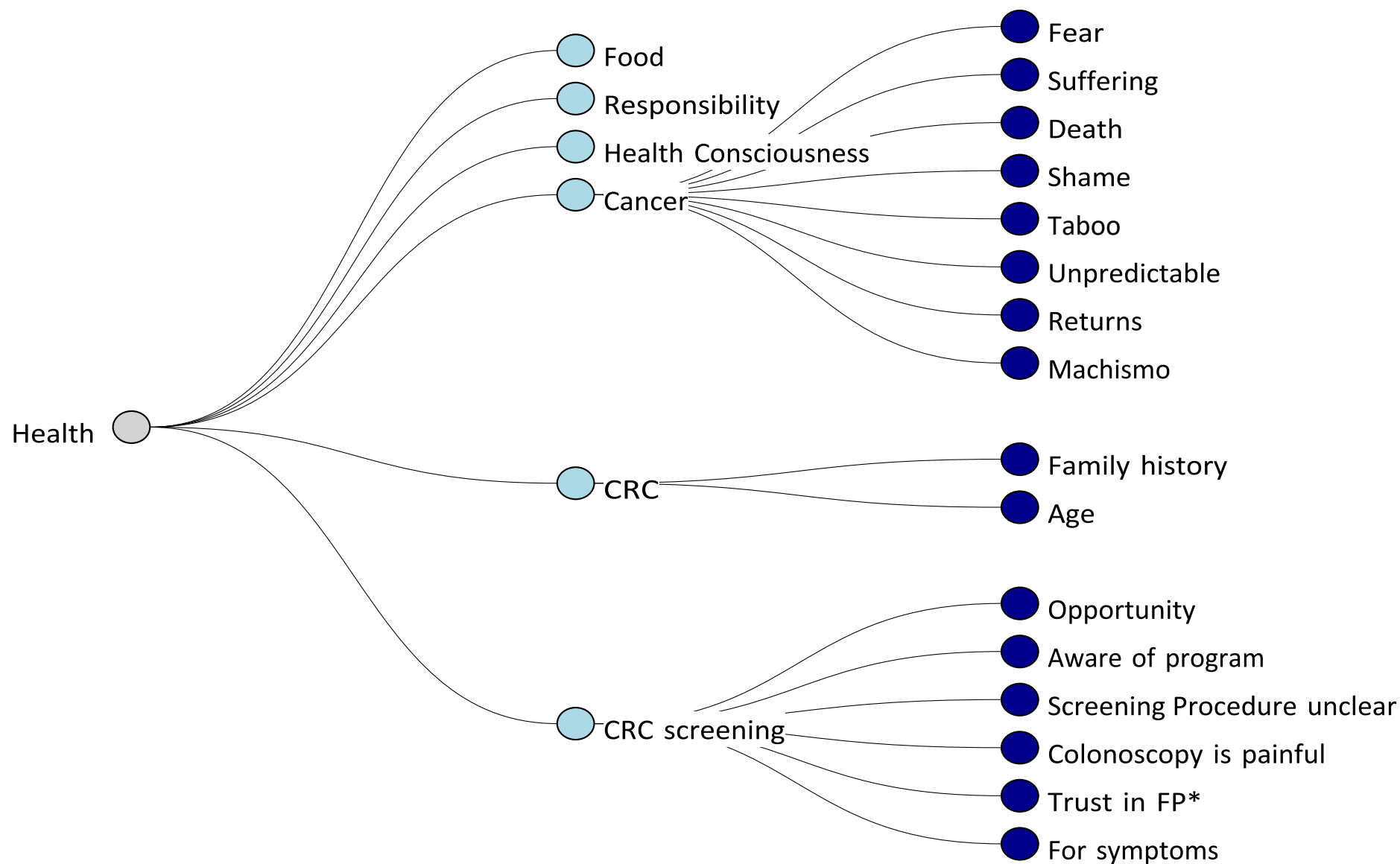

Supplement: Supplementary file 3 — Supplementary Material 3 [file 12889_2023_16335_MOESM3_ESM.pdf]

**Supplementary file 4**

**Interview themes about decision-making**

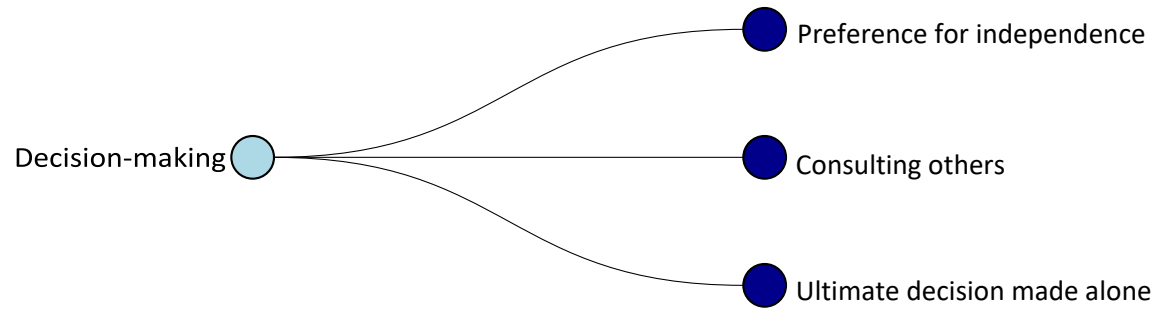

Supplement: Supplementary file 4 — Supplementary Material 4 [file 12889_2023_16335_MOESM4_ESM.pdf]

## Supplementary file 5

### Interview themes about information

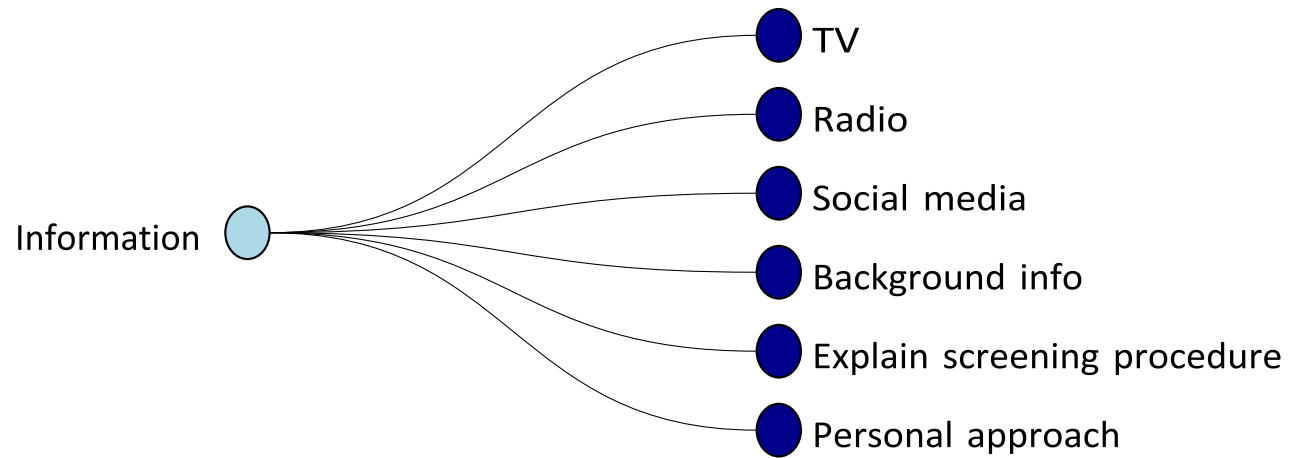

Supplement: Supplementary file 5 — Supplementary Material 5 [file 12889_2023_16335_MOESM5_ESM.pdf]
